# Assessing the Effects of Thymol and Oxalic Acid on Honey Bee Colony Condition Using Ratiometric Spectral Indicators in Honey and Beeswax

**DOI:** 10.3390/insects17040440

**Published:** 2026-04-21

**Authors:** Mira Stanković, Miroslav Nikčević, Sladjana Z. Spasić, Ksenija Radotić

**Affiliations:** 1 Institute for Multidisciplinary Research, National Institute of the Republic of Serbia, University of Belgrade, Kneza Višeslava 1, 11030 Belgrade, Serbia; mira.mutavdzic@imsi.bg.ac.rs (M.S.); mvnikcevic@imsi.bg.ac.rs (M.N.); sladjana@imsi.rs (S.Z.S.); 2 Center for Green Technologies, Institute for Multidisciplinary Research, University of Belgrade, Kneza Višeslava 1, 11000 Belgrade, Serbia

**Keywords:** *Apis mellifera*, *Varroa destructor*, honey, beeswax, phenolics, proteins

## Abstract

Over the past 20 years, honey bee colony declines have been driven by multiple factors, notably diseases and parasites. The parasitic mite *Varroa destructor*, which weakens the bees’ immune systems, has been particularly harmful. While various synthetic acaricides are used, the chemicals may accumulate in the beeswax, endangering colony health and allowing *Varroa* populations to develop resistance to these acaricides. These problems have prompted interest in organic alternatives like thymol and oxalic acid. In this study, colony health was assessed through the proteins-to-phenolics spectral ratio in honey and beeswax, determined by fluorescence spectroscopy, as a ratiometric indicator of infection level in treated hives. Over two months, hives were treated with either oxalic acid, thymol, or remained untreated as controls. Neither treatment significantly affected the proteins-to-phenolics ratios in honey, ranging from 0.30 to 0.83, or in beeswax, ranging from 1.40 to 1.83, suggesting that the incorporation of these vital constituents remains stable despite acaricide application. While thymol demonstrates potential adverse effects on bee health, careful management of treatment concentrations is essential to ensure both the efficacy of *Varroa* control and the preservation of honey quality. These findings provide valuable insights for beekeepers regarding the safe application of organic acaricides.

## Simple Summary

Honey bee colonies are declining worldwide, largely due to the parasitic mite *Varroa destructor*, which weakens bees and spreads diseases. Synthetic chemicals used to control these mites can accumulate in beeswax and may harm colonies or lead to resistant mite populations. Therefore, natural treatments such as thymol and oxalic acid are increasingly used. In this study, we applied fluorescence spectroscopy to monitor the ratio of protein and phenolic compounds in honey as an indicator of colony condition during a two-month treatment period. Our results show that the incorporation of these important natural constituents in honey remains stable despite treatment with the organic acaricides oxalic acid and thymol. These findings indicate that oxalic acid and thymol can help control mites while preserving honey quality, supporting healthy bee colonies essential for pollination and food production.

## 1. Introduction

Honey bees (*Apis mellifera* L.) are essential to agriculture as pollinators. The significant decline in honey bee populations over the past two decades threatens crop production and apiculture sustainability [1,2,3]. A major driver of this decline is the parasitic mite *Varroa destructor* (Mesostigmata: Varroidae), which weakens bee immunity and transmits viruses [4]. Synthetic acaricides such as coumaphos, tau-fluvalinate, and amitraz are widely used for mite control, but leave residues in beeswax and honey, which both negatively affects bee health and leads to an increase in mite resistance [5,6,7,8,9,10].

Natural alternatives to synthetic acaricides, particularly oxalic acid and thymol, are therefore commonly used. Oxalic acid is naturally present in honey, whereas thymol, derived from *Thymus vulgaris* and other plants, is commonly used in beekeeping [11,12]. Thymol has shown variable efficacy against *Varroa destructor* [13,14,15,16] and *Nosema ceranae* (Microsporida (1MCRPO), family Nosematidae (1NOSMF)) while also enhancing bees’ immune response and reducing spore load [16]. However, thymol may leave residues in hive products [17,18] and alter honey taste [19,20]. At higher concentrations, it may negatively affect bee oxidative capacity, and potentially induces genotoxic effects [21]. In contrast, oxalic acid treatments generally appear to have no adverse effects on bees or brood [22], and their use does not significantly alter the levels of oxalic acid present in honey [23]. Oxalic acid may also be used in combination with other acaricides, both natural and synthetic, for increased efficacy against *Varroa destructor* while maintaining colony stability [24].

Fluorescence spectroscopy is a rapid and non-destructive method of analyzing the biochemical composition of various types of materials [25,26,27,28,29,30,31,32,33,34]. Proteins and phenolic compounds, although minor constituents of honey, are key intrinsic fluorophores [25,26,27,34]. Phenolics, in honey, originate mainly from plant nectar, while proteins derive predominantly from bees and, in small part, from pollen [35]. Previous studies have demonstrated that the proteins-to-phenolics ratio derived from fluorescence emission spectra of honey was in agreement with spectrophotometric quantification [25] and shows a negative linear correlation with infestation levels of *V. destructor* and *N. ceranae* [27].

Previous uses of fluorescence spectroscopy have addressed biochemical markers in honey, particularly the proteins-to-phenolics balance and its relationship with colony health [25,26,27,34]. There is limited knowledge on how commonly used naturally occurring acaricides, such as oxalic acid and thymol, directly influence fluorescence-derived compositional markers in honey, particularly the proteins-to-phenolics balance. Since the proteins-to-phenolics ratio can reflect a colony’s physiological status and pathogen burden, understanding its response to acaricide treatment is important for evaluating the potential sublethal effects of acaricides on bee health. The aim of the present study was to apply fluorescence spectroscopy not only as a diagnostic tool for colony condition, but also as a means of detecting treatment-induced changes in honey and beeswax biochemical markers, thereby linking acaricide application with potential impacts on both bee physiology/health and honey quality. Specifically, the study evaluated the response of a previously validated indicator (proteins-to-phenolics ratio) in honey under conditions of acaricide treatment routinely applied in beekeeping practice. To determine how common natural acaricides influence fluorescence-derived compositional markers in honey, this study examines the effects of a two-month treatment with oxalic acid or thymol on biochemical markers in honey using fluorescence spectroscopy. The relative quantities of proteins and phenolics were estimated from honey fluorescence emission spectra, and their ratio was used as an indicator of treatment-induced variability in these biochemical markers. Additionally, beeswax was analyzed as a potential site of residue accumulation and compositional changes. The results provide insight into the effects of thymol and oxalic acid treatments on honey bee health as well as the use of honey emission spectra to monitor treatment-induced changes in biochemical markers. These findings contribute to understanding how this approach can be used to evaluate the suitability of these acaricide treatments in sustainable beekeeping practices.

## 2. Materials and Methods

The test apiary, located in the field Oduševac, Stari Slankamen, Serbia (45°04′48″ N, 20°17′09″ E, 539 m altitude), contained ten hives. Nucleus colonies (nucs) were purchased in May 2020. Weather conditions during the sampling period were typical for this region; monthly precipitation fell within the long-term average range (approximately 40–100 mm depending on the month), and temperatures were in line with seasonal temperature patterns, including higher temperatures in July–August (maximum 32–34 °C), supporting normal nectar flow conditions. During the first two weeks of June, the hives were not treated with any substances, and honey and beeswax samples were collected on two occasions to observe the condition of the apiary. Although no visual evidence of *Varroa* was observed by the experienced beekeeper during the pre-treatment sampling period, it is assumed that the mite was present in the apiary.

To avoid positional bias, ten hives were selected, and each hive was randomly assigned to one of three groups: a control group (*n* = 4), an oxalic acid treatment group (*n* = 3), and a thymol treatment group (*n* = 3). To control for potential differences in the hives’ baseline states, and thus account for potential variations in the post-treatment samples, honey and beeswax samples were collected from each hive on two occasions prior to treatment (7 June and 14 June 2020), as mentioned above. These measurements were used as baseline controls.

**Figure 1:**
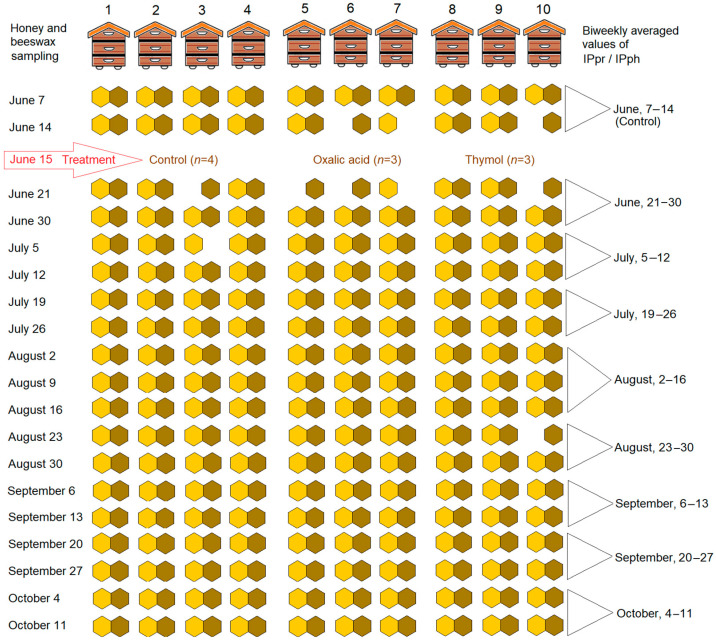
The experimental design outlines the time frame for treatments, sampling methods, and sample size. Each hive serves as a single experimental unit (*n* = 10). In the above diagram, each hexagon represents a honey (yellow) or beeswax (ochre) sample. A missing hexagon indicates that a sample could not be collected. Honey and beeswax samples were gathered on designated sampling dates (shown on the left) and subsequently analyzed using spectroscopy. The proteins to phenolics ratio (IPpr/IPph) was calculated for each sample, with these values averaged every two weeks for each of the three groups. In August, there were five sampling dates, and the average was based on three measurements (indicated by triangles on the right).

The treatment duration was 17 weeks, from 15 June to 11 October 2020. Three hives were treated with oxalic acid, three with Apiguard (25% thymol) (Laleham Health and Beauty Limited, Alton, UK), and four served as controls (Figure 1). A total of 183 honey samples and 187 beeswax samples were collected from the ten hives used as experimental units (*n* = 10). Oxalic acid treatment involved placing shop towels soaked with a 1:1:1 oxalic acid, glycerin, and water solution across the brood box top bars (36 g per hive). Apiguard trays were placed on brood frames and replaced every 28 days. All honey samples were multifloral.

Figure 1 shows the experimental plan—the timeline for treatments and sampling as well as the sample size. Beginning on 7 June 2020, honey and beeswax samples were collected weekly from untreated (control) and treated hives. Honey was collected from uncapped cells, and beeswax was obtained from a nearby new comb. The honey samples were taken by syringe from the uncapped cells of the same frame each week, located in the first box of each hive. If only capped cells were present on the frame, samples were instead collected from the nearest frame that contained uncapped cells. The beeswax samples were collected from the newly made beeswax at the same location as the honey sample or the nearest available place. Samples were stored in 1.5 mL tubes at room temperature in the dark.

Fluorescence spectra of honey and beeswax samples were recorded using an Fl3-221 P spectrofluorometer (Jobin Yvon, Horiba, Palaiseau, France) with a 450 W Xe lamp and photomultiplier tube. Samples were placed in a solid holder with Rayleigh masking applied to reduce scattering. Emission spectra (275–500 nm) were measured after 260–380 nm excitation, with an integration time of 0.1 s. Slits were 1 nm for honey and 0.5 nm for beeswax samples. Spectra were measured at varying excitation wavelengths and averaged to capture all fluorophore contributions.

The ratio of the peak intensity of the emission for the protein and phenolic spectral components (IPpr/IPph) was calculated for each sample. The obtained IPpr/IPph values were then averaged biweekly, separately for each treatment group and the control group (see Figure 1) [25,26]. In June, July, and September, there were four sampling dates per month, and each biweekly average ratio was thus the average of values obtained from two sampling dates. In August, there were five sampling dates, and the average for the first half of that month thus took into account measurements from three instead of two sampling dates. The schedule of sampling and calculating average ratios is indicated by the triangles on the right side of Figure 1.

Exploratory and data analyses were performed using IBM SPSS Statistics 27 software (IBM, Armonk, NY, USA). The transformed data IPpr/IPph, calculated from the emission spectra in different treatments, were used as input variables. The number of observations (hives) was four (*n* = 4) for the control and three (*n* = 3) for each treatment in 9 time periods (Figure 1).

For each hive, values obtained at the two pre-treatment sampling points were averaged to obtain a single baseline value, at the following time point: 1–14 June, Control (Figure 1). Differences in baseline IPpr/IPph ratios between the three groups were evaluated using the Kruskal–Wallis non-parametric test at α = 0.05.

Due to the small sample size and the relatively large number of repeated measurements, a linear mixed-effects model (LMM) was used to analyze the effects of treatment and time on IPpr/IPph ratios in honey and beeswax. Each hive serves as a single experimental unit/subject (*n* = 10). Treatment (control, oxalic acid, thymol), time (nine sampling points), and their interaction were included as fixed effects, while subject was included as a random effect to account for repeated measurements. An appropriate covariance structure (autoregressive, AR(1)) was selected based on model fit. Post hoc comparisons between treatments and controls were performed using Bonferroni adjustments for multiple comparisons (α = 0.05). Degrees of freedom were estimated using the Satterthwaite approximation. When dates are written as intervals (e.g., 7–14 June), the corresponding values represent averages of the measurements obtained on the two specified dates.

## 3. Results

No decline of bee population according to seasonal variation was observed during treatment with oxalic acid or thymol, indicating that neither treatment had any harmful effect on the bees.

**Figure 2:**
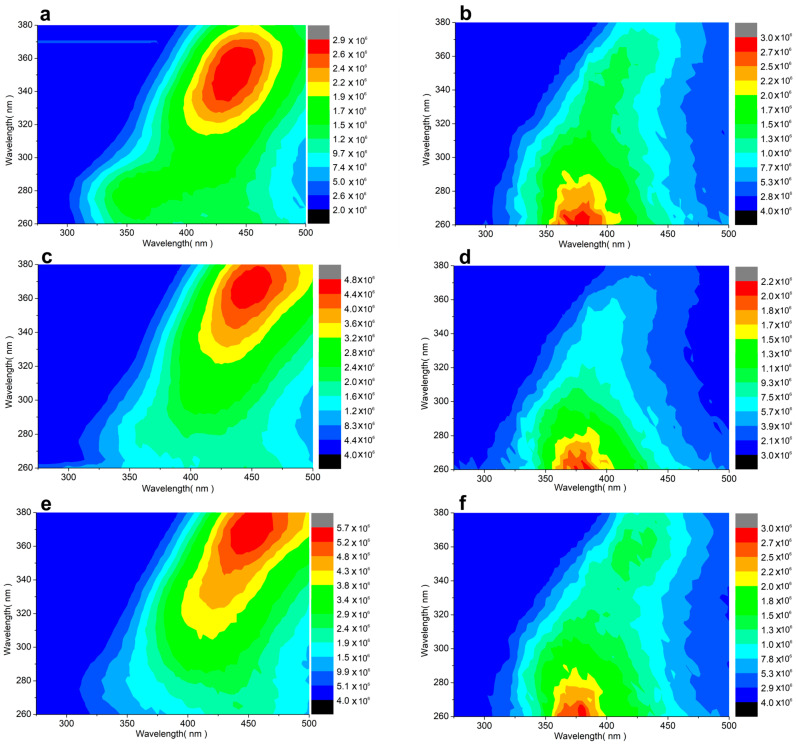
Fluorescence EEM contour maps of honey and beeswax samples from the three hive groups, as follows: (**a**) honey, control, (**b**) beeswax, control, (**c**) honey, after oxalic acid treatment, (**d**) beeswax, after oxalic acid treatment, (**e**) honey, after thymol treatment, and (**f**) beeswax, after thymol treatment. X and Y axes show emission and excitation wavelengths, respectively. False colors indicate fluorescence intensity (blue = low, red = high).

Figure 2 shows the fluorescence Excitation–Emission (EEM) contour maps for multifloral honey and beeswax from control (a,b), oxalic acid-treated (c,d), and thymol-treated (e,f) hives. The emission spectra, ranging from 300 to 500 nm, are primarily from amino acids/proteins and phenolics [36].

We determined the ratio of proteins-to-phenolics spectral components, as the ratio of emission peak intensities at the amplitude (maximum) for the proteins and phenolics spectral components (IPpr/IPph). In our previous work, it was shown that the ratio of protein to phenolic components in the honey emission spectra matched their ratio determined by spectrophotometric (colorimetric) quantification [25].

**Figure 3:**
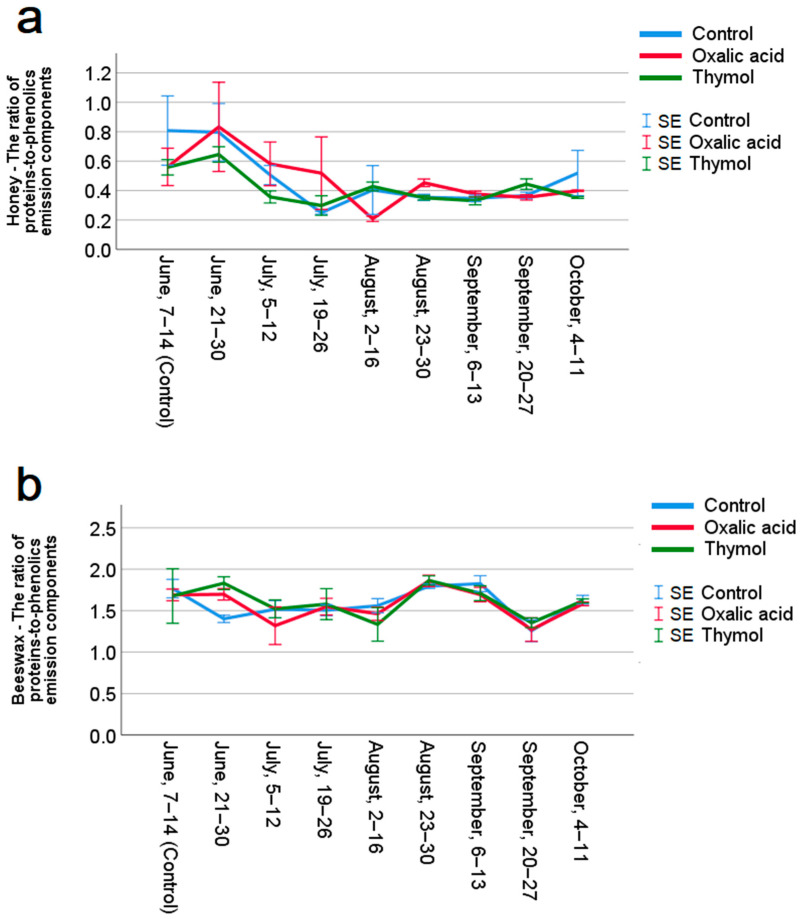
Mean ratios of proteins-to-phenolics emission components (IPpr/IPph) (±SE) in the emission spectra of the honey (**a**) and beeswax (**b**) samples, across treatments and sampling times. The IPpr/IPph values obtained were then averaged biweekly for each treated group and the control group separately. Results of the linear mixed-effects model indicated a significant effect of time, with a *p* < 0.01 for honey and *p* < 0.001 for beeswax. However, neither the treatment nor the interaction between treatment and time was found to be significant in the analyses of both honey and beeswax. Bonferroni-adjusted post hoc comparisons of both honey and beeswax indicated that none of the ratios obtained from the post-treatment sampling time points differed significantly from the pre-treatment measurements (7–14 June, control).

Figure 3 shows the IPpr/IPph ratio for honey (a) and beeswax (b) plotted against the nine time points (June to October 2020) when the samples were collected from the corresponding hives. At each time point, the averaged values of IPpr/IPph from 3–4 hives per group are shown. The proteins-to-phenolics ratio varied between 0.30 and 0.83 for honey, and between 1.40 and 1.83 for beeswax, across different samples.

No significant differences in baseline IPpr/IPph ratios were detected among the control, oxalic acid, and thymol associated groups (Kruskal–Wallis H(2) = 0.12, *p* = 0.943). Monte Carlo estimation (10,000 samples) confirmed this result (*p* = 0.971), indicating that the groups were comparable before treatment (Supplementary Figure S1).

A linear mixed-effects model revealed a significant effect of time on the proteins-to-phenolics ratios (IPpr/IPph) in honey, F(8, 37.257) = 3.371, *p* = 0.005. However, the main effect of treatment was not significant, F(2, 12.996) = 0.397, *p* = 0.680, nor was the interaction between treatment and time significant, F(16, 37.257) = 0.867, *p* = 0.608. These results suggest that while IPpr/IPph ratios varied over time, they were not significantly influenced by the treatment. Pairwise comparisons assessing the differences in IPpr/IPph ratios over time revealed no significant changes when compared to pre-treatment values, such as those from 7–14 June in the control group.

Similarly, in beeswax, a significant effect of time on IPpr/IPph ratios was observed, F(8, 33.850) = 6.392, *p* < 0.001. Again, the main effect of treatment was not significant, F(2, 22.051) = 0.276, *p* = 0.762, nor was the interaction between treatment and time significant, F(16, 33.850) = 0.815, *p* = 0.660. Post hoc comparisons regarding the IPpr/IPph ratios over time showed no significant changes compared to the pre-treatment values in beeswax.

## 4. Discussion

The variation in IPpr/IPph ratios over time suggests that changes are likely driven by natural fluctuations over the sampling period rather than by the applied treatments. Such variability may reflect seasonal dynamics, environmental conditions, or intrinsic changes in hive composition, have been reported [37].

Importantly, no significant differences in baseline IPpr/IPph ratios were observed among the three experimental groups before treatment, confirming that the groups were comparable at the outset and that no initial bias was introduced during group allocation. This supports the validity of the applied linear mixed-effects model and strengthens confidence in the conclusion that the apparent lack of treatment effect on IPpr/IPph ratios is not attributable to pre-existing differences among groups.

It was previously shown that the genotoxic effects of thymol on cultured honey bee cells depend on the applied concentration [24]. Another study assessed the effects of different acaricides on the gene expression profile comprising genes acting on diverse metabolic levels (detoxification, immunity, and development), applying the treatments to hives in which the honey bee populations lacked the influence of *Varroa* mites. The results indicate that thymol may alter some metabolic responses, including detoxification gene expression pathways, components of the immune system responsible for cellular response, and developmental genes. Such effects could be harmful to the health of individual honey bees and entire colonies [37]. Thymol was found to accumulate in the bodies and brains of honeybees after 24 h of chronic exposure in the hives, and it was also found to stay in the beeswax [38]. Recent experimental studies have demonstrated that while higher doses of oxalic acid can significantly suppress mite populations, they may negatively affect honey bee brood development, indicating a trade-off between acaricide efficacy and honey bee safety [39]. In addition, emerging evidence suggests that oxalic acid treatments can influence honey bee microbiota, with some studies reporting reductions in pathogen load and shifts toward beneficial bacterial communities, although these effects appear to depend more on environmental and management factors than on the treatment itself [40].

Direct comparison between studies of the effects of oxalic acid and thymol on honey bees is difficult as the application methods and dosages employed have not been consistent in the published literature. In the present study, the dosages and application protocols used were those that reflect common beekeeping practice [23,41]. Antivarroa treatments with oxalic acid or thymol did not significantly alter the protein/phenolic ratio in honey or beeswax compared with the corresponding control. This finding suggests that these treatments do not markedly influence the incorporation of protein and phenolic compounds into hive products under the conditions tested. In addition, the absence of significant seasonal variations in protein and phenolic content points to consistent foraging activity throughout the study period. Proper thymol or oxalic acid use ensures colony health, sustaining honey production, market value, and beekeeper profits. Although thymol may have some adverse effects on bees, it does not affect the crucial protein and phenolic composition of honey. The findings are relevant to the tested concentrations, which are effective against *Varroa destructor* while maintaining the integrity of honey composition. Beekeepers should carefully select appropriate concentrations of thymol or oxalic acid for hive treatments to protect colony health and honey quality.

The results obtained align with previous studies emphasizing the importance of using naturally occurring compound-based treatments in beekeeping, as these treatments offer an effective alternative to synthetic acaricides, which may leave harmful residues in hive products and negatively affect bee health. Given increasing consumer demand for natural and residue-free honey, our study further supports the preference for oxalic acid and thymol as sustainable *Varroa* control options.

## 5. Conclusions

Fluorescence spectroscopy, a sensitive, rapid, and non-destructive method, proved particularly suitable for this study due to its ability to analyze large numbers of samples without the need for preprocessing. Its effectiveness in detecting protein and phenolic components in honey and beeswax renders it a valuable tool for monitoring hive product quality following acaricide treatments, thereby supporting both apicultural health management and commercial quality standards. However, despite these methodological advantages, no significant treatment effects on IPpr/IPph ratios were detected. The absence of detectable effects may be attributed to several factors, including the relatively small sample size and inherent biological variability, which can limit statistical power. It is also possible that the applied treatments did not induce changes of sufficient magnitude to be distinguishable from natural variation over time. Future research should therefore aim to expand on these findings by incorporating larger sample sizes, extending observations across multiple seasons, and exploring additional honey constituents that may respond to *Varroa* control strategies.

## Data Availability

The raw data supporting the conclusions of this article will be made available by the authors on request.

## Supplementary Materials

The following supporting information can be downloaded at: https://www.mdpi.com/article/10.3390/insects17040440/s1, Figure S1. The mean ratios of proteins-to-phenolics emission components (IPpr/IPph) (±SE) in the fluorescence spectra of honey (a) and beeswax (b) samples were measured in the baseline control across different treatment groups. The values for IPpr/IPph obtained from the pre-treatment measurements conducted on 7 June and 14 June were averaged and labeled as “7–14 June Control” for each treatment and control group separately. No significant differences in baseline IPpr/IPph ratios were found among the control, oxalic acid, and thymol treatment groups (Kruskal–Wallis H(2) = 0.12, *p* = 0.943). A Monte Carlo estimation with 10,000 samples confirmed these results (*p* = 0.971), indicating that the groups were comparable before treatment.

## References

[1] Kortsch S., Timberlake T.P., Cirtwill A.R., Sapkota S., Rokoya M., Devkota K., Roslin T., Memmott J., Saville N. (2024). Decline in honeybees and its consequences for beekeepers and crop pollination in western Nepal. Insects.

[2] Potts S.G., Roberts S.P., Marris G., Brown M.A., Jones R., Neumann P., Settele J. (2010). Declines of managed honey bees and beekeepers in Europe. J. Apic. Res..

[3] VanEngelsdorp D., Meixner M.D. (2010). A historical review of managed honey bee populations in Europe and the United States and the factors that may affect them. J. Invertebr. Pathol..

[4] Haber A.I., Steinhauer N.A., VanEngelsdorp D. (2019). Use of chemical and nonchemical methods for the control of *Varroa destructor* (Acari: Varroidae) and associated winter colony losses in U.S. beekeeping operations. J. Econ. Entomol..

[5] Lozano A., Hernando M.D., Uclés S., Hakme E., Fernández-Alba A.R. (2019). Identification and measurement of veterinary drug residues in beehive products. Food Chem..

[6] Murcia Morales M., Gómez Ramos M.J., Parrilla Vázquez P., Díaz Galiano F.J., García Valverde M., Gámiz López V., Flores J.M., Fernández-Alba A.R. (2020). Distribution of chemical residues in the beehive compartments and their transfer to the honeybee brood. Sci. Total Environ..

[7] Traynor K.S., Pettis J.S., Tarpy D.R., Mullin C.A., Frazier J.L., Frazier M., VanEngelsdorp D. (2016). In-hive pesticide exposome: Assessing risks to migratory honey bees from in-hive pesticide contamination in the Eastern United States. Sci. Rep..

[8] Tsigouri A.D., Menkissoglu-Spiroudi U., Thrasyvoulou A. (2001). Study of tau-fluvalinate persistence in honey. Pest Manag. Sci..

[9] Damiani N., Maggi M.D., Gende L.B., Faverin C., Eguaras M.J., Marcangeli J.A. (2010). Evaluation of the toxicity of a propolis extract on *Varroa destructor* (Acari: Varroidae) and *Apis mellifera* (Hymenoptera: Apidae). J. Apic. Res..

[10] Medici S.K., Maggi M.D., Sarlo E.G., Ruffinengo S., Marioli J.M., Eguaras M.J. (2015). The presence of synthetic acaricides in beeswax and its influence on the development of resistance in *Varroa destructor*. J. Apic. Res..

[11] Maistrello L., Lodesani M., Costa C., Leonardi F., Marani G., Caldon M., Mutinelli F., Granato A. (2008). Screening of natural compounds for the control of Nosema disease in honeybees (*Apis mellifera*). Apidologie.

[12] Kovacevic Z., Kladar N., Cabarkapa I., Radinovic M., Maletic M., Erdeljan M., Bozin B. (2021). New perspective of *Origanum vulgare* L. and *Satureja montana* L. essential oils as bovine mastitis treatment alternatives. Antibiotics.

[13] Chiesa F., D’Agaro M. (1991). Effective control of varroatosis using powdered thymol. Apidologie.

[14] Imdorf A., Kilchenmann V., Bogdanov S., Bachofen B., Beretta C. (1995). Toxicity of thymol, camphor, menthol, and eucalyptus to *Varroa jacobsoni* Oud and *Apis mellifera* L. in laboratory tests. Apidologie.

[15] Stanimirovic Z., Glavinic U., Ristanic M., Aleksic N., Jovanovic N.M., Vejnovic B., Stevanovic J. (2019). Looking for the causes of and solutions to the issue of honey bee colony losses. Acta Vet. Beogr..

[16] Glavinic U., Blagojevic J., Ristanic M., Stevanovic J., Lakic N., Mirilovic M., Stanimirovic Z. (2022). Use of thymol in *Nosema ceranae* control and health improvement of infected honey bees. Insects.

[17] Tihelka E. (2018). Effects of synthetic and organic acaricides on honey bee health: A review. Slov. Vet. Res..

[18] Manzano Sánchez L., Gómez Ramos M.J., Gómez-Ramos M.D., Parrilla Vázquez P., Flores J.M., Fernández-Alba A.R. (2021). Presence, persistence and distribution of thymol in honeybees and beehive compartments by high resolution mass spectrometry. Environ. Adv..

[19] Gao H., Cao W., Liang Y., Cheng N., Wang B.N., Zheng J.B. (2010). Determination of thymol and phenol in honey by LC with electrochemical detection. Chromatographia.

[20] Tonello N., Moressi M.B., Robledo S.N., D’Eramo F., Marioli J.M. (2016). Square wave voltammetry with multivariate calibration tools for determination of eugenol, carvacrol and thymol in honey. Talanta.

[21] Glavinic U., Rajkovic M., Ristanic M., Stevanovic J., Vejnovic B., Djelic N., Stanimirovic Z. (2023). Genotoxic potential of thymol on honey bee DNA in the comet assay. Insects.

[22] Berry J.A., Bartlett L.J., Bruckner S., Baker C., Braman S.K., Delaplane K.S., Williams G.R. (2022). Assessing repeated oxalic acid vaporization in honey bee (Hymenoptera: Apidae) colonies for control of the ectoparasitic mite *Varroa destructor*. J. Insect Sci..

[23] Maggi M., Tourn E., Negri P., Szawarski N., Marconi A., Gallez L., Medici S., Ruffinengo S., De Feudis L., Quintana S. (2016). A new formulation of oxalic acid for *Varroa destructor* control applied in *Apis mellifera* colonies in the presence of brood. Apidologie.

[24] Ristanić M., Glavinić U., Stevanović J., Cvetković T., Mijatović A., Vejnović B., Stanimirović Z. (2025). Formic Acid-Based Preparation in *Varroa destructor* Control and Its Effects on Hygienic Behavior of *Apis mellifera*. Insects.

[25] Stanković M., Bartolić D., Šikoparija B., Spasojević D., Mutavdžić D., Natić M., Radotić K. (2019). Variability estimation of the protein and phenol total content in honey using front face fluorescence spectroscopy coupled with MCR–ALS analysis. J. Appl. Spectrosc..

[26] Stanković M., Prokopijević M., Šikoparija B., Nedić N., Andrić F., Polović N., Natić M., Radotić K. (2023). Using front-face fluorescence spectroscopy and biochemical analysis of honey to assess a marker for the level of *Varroa destructor* infestation of honey bee (*Apis mellifera*) colonies. Foods.

[27] Stanković M., Bartolić D., Mutavdžić D., Marković S., Grubić S., Jovanović N.M., Radotić K. (2023). Estimation of honey bee colony infection with *Nosema ceranae* and *Varroa destructor* using fluorescence spectroscopy in combination with differential scanning calorimetry of honey samples. J. Apic. Res..

[28] Bartolić D., Stanković M., Mutavdžić D., Stanković S., Jovanović D., Radotić K. (2018). Multivariate curve resolution–alternate least square analysis of excitation-emission matrices for maize flour contaminated with aflatoxin B1. J. Fluoresc..

[29] Bartolić D., Stanković M., Prokopijević M., Radotić K. (2022). Effects of UV-A and UV-B irradiation on antioxidant activity and fluorescence characteristics of soybean (*Glycine max* L.) seeds. Russ. J. Phys. Chem. A.

[30] Brdar S., Panić M., Matavulj P., Stanković M., Bartolić D., Šikoparija B. (2023). Explainable AI for unveiling deep learning pollen classification model based on fusion of scattered light patterns and fluorescence spectroscopy. Sci. Rep..

[31] Mitrović A.L.J., Simonović Radosavljević J., Prokopijević M., Spasojević D., Kovačević J., Prodanović O., Todorović B., Matović B., Stanković M., Maksimović V. (2021). Cell wall response to UV radiation in needles of *Picea omorika*. Plant Physiol. Biochem..

[32] Nikolić D., Kostić J., Đorđević Aleksić J., Sunjog K., Rašković B., Poleksić V., Pavlović S., Borković-Mitić S., Dimitrijević M., Stanković M. (2024). Effects of mining activities and municipal wastewaters on element accumulation and integrated biomarker responses of the European chub (*Squalius cephalus*). Chemosphere.

[33] Radotić K., Stanković M., Bartolić D., Natić M. (2023). Intrinsic fluorescence markers for food characteristics, shelf life, and safety estimation: Advanced analytical approach. Foods.

[34] Stanković M., Nikčević M., Radotić K. (2020). Annual variation of proteins and phenols in honey of a bee society using fluorescence spectroscopy: A way to assess effects of antivarroa treatments on honey composition. Eur. Food Res. Technol..

[35] Nazarian H., Taghavizad R., Majd A. (2010). Origin of honey proteins and method for its quality control. Pak. J. Bot..

[36] Ruoff K., Luginbühl W., Künzli R., Bogdanov S., Bosset J.O., Von der Ohe K., Von der Ohe W., Amado R. (2006). Authentication of the botanical and geographical origin of honey by front-face fluorescence spectroscopy. J. Agric. Food Chem..

[37] Boncristiani H., Underwood R., Schwarz R., Evans J.D., Pettis J., VanEngelsdorp D. (2012). Direct effect of acaricides on pathogen loads and gene expression levels in honey bees (*Apis mellifera*). J. Insect Physiol..

[38] Carayon J.L., Téné N., Bonnafé E., Alayrangues J., Hotier L., Armengaud C., Treilhou M. (2014). Thymol as an alternative to pesticides: Persistence and effects of Apilife Var on the phototactic behavior of the honeybee (*Apis mellifera*). Environ. Sci. Pollut. Res..

[39] Bozkus M., Breece C., Lucas H., Steinhauer N.A., Sagili R.R. (2025). Oxalic Acid Vaporization: Effectiveness against *Varroa destructor* (Mesostigmata: Varroidae) and Safety for *Apis mellifera* (Hymenoptera: Apidae). J. Insect Sci..

[40] Gorrochategui-Ortega J., Muñoz-Colmenero M., Galartza E., Estonba A., Zarraonaindia I. (2025). Colonies under Dysbiosis Benefit from Oxalic Acid Application: The Role of Landscape and Beekeeping Practices in Microbiota Response to Treatment. J. Pest Sci..

[41] Oliver R. (2018). ScientificBeekeeping.com. https://scientificbeekeeping.com/.

