# Supplementary material for: Assessing the Effects of Thymol and Oxalic Acid on Honey Bee Colony Condition Using Ratiometric Spectral Indicators in Honey and Beeswax
Source: Insects. 2026 Apr 21;17(4):440. doi: 10.3390/insects17040440 (PMC13117002; doi:10.3390/insects17040440)
Supplement: Supplementary file 1 [file insects-17-00440-s001.zip › insects-4221621-supplementary.pdf]

Assessing the Effects of Thymol and Oxalic Acid on Honey Bee Colony Condition Using Ratiometric Spectral Indicators in Honey and Beeswax

Mira Stanković [1,2], Miroslav Nikčević [1], Sladjana Z. Spasić [1], Ksenija Radotić [1,2,*]

[1] Institute for Multidisciplinary Research, National Institute of the Republic of Serbia, University of Belgrade, Kneza Višeslava 1, 11030 Belgrade, Serbia;

[2] Center for Green Technologies, Institute for Multidisciplinary Research, University of Belgrade, Kneza Višeslava 1, 11000 Belgrade, Serbia

* Correspondence: xenia@imsi.bg.ac.rs

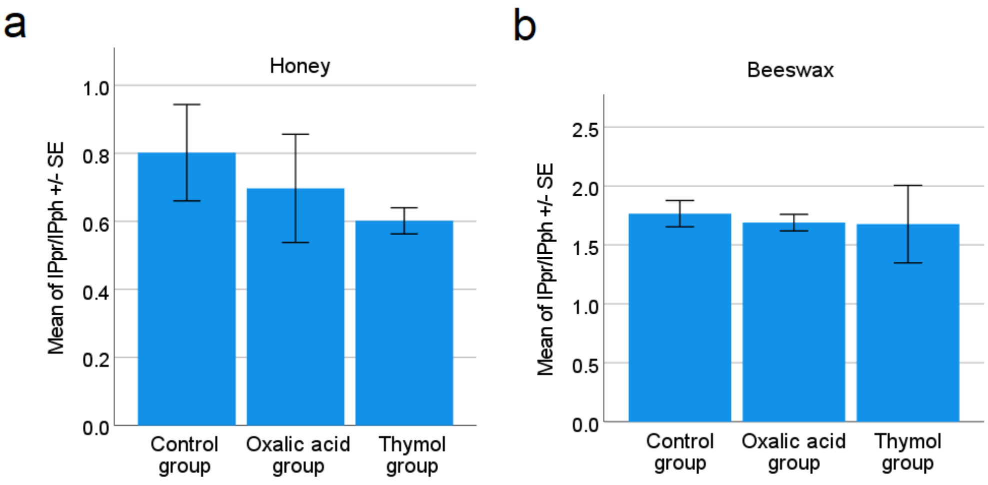

Figure S1. The mean ratios of proteins-to-phenolics emission components (IPpr/IPph) (±SE) in the fluorescence spectra of honey (a) and beeswax (b) samples were measured in the baseline control across different treatment groups. The values for IPpr/IPph obtained from the pre-treatment measurements conducted on 7 June and 14 June were averaged and labeled as "7–14 June Control" for each treatment and control group separately. No significant differences in baseline IPpr/IPph ratios were found among the control, oxalic acid, and thymol treatment groups (Kruskal–Wallis $H(2) = 0.12$, $p = 0.943$). A Monte Carlo estimation with 10,000 samples confirmed these results ($p = 0.971$), indicating that the groups were comparable before treatment.
